# Tertiary lymphoid structures in breast cancer: formation, immune functions and clinical implications

**DOI:** 10.3389/fimmu.2026.1827875

**Published:** 2026-06-02

**Authors:** Wenting Dong, Shichao Ai, Xiaofei Shen, Yongzhong Yao

**Affiliations:** 1 Division of Breast Surgery, Department of General Surgery, Nanjing Drum Tower Hospital, School of Medicine, Southeast University, Nanjing, China; 2 Division of Gastric Surgery, Department of General Surgery, Nanjing Drum Tower Hospital, the Affiliated Hospital of Nanjing University Medical School, Ministry of Education (MOE) Key Laboratory of Model Animal for Disease Study, Nanjing, China

**Keywords:** breast cancer, immune checkpoint blockade, immunotherapy, prognostic biomarkers, tertiary lymphoid structures, tumor immunity, tumor microenvironment

## Abstract

Tertiary lymphoid structures (TLS) are ectopic lymphoid aggregates that arise in non-lymphoid tissues under chronic inflammatory conditions and during tumor development. Although TLS have often been associated with a favorable prognosis and enhanced therapeutic responsiveness, their biological and clinical significance in breast cancer is highly heterogeneous and cannot be adequately captured by a simple TLS-positive versus TLS-negative classification. In this review, we summarize the structural organization and developmental stages of TLS, with an emphasis on the functional relevance of maturation, including germinal center formation. We further discuss current concepts regarding the mechanisms driving TLS initiation and maturation, including stromal activation, chemokine-guided immune cell recruitment, and high endothelial venule-mediated lymphocyte trafficking. In addition, we examine the major dimensions of TLS heterogeneity in breast cancer, including differences in maturity, spatial location, molecular subtype, and immune cellular composition, and discuss how these variables shape antitumor, immunoregulatory, and context-dependent TLS functions. We also evaluate the current approaches for TLS identification and classification, highlighting the strengths and limitations of histopathology, immunophenotyping, multiplex imaging, and transcriptomic inference. Finally, we discuss the prognostic and predictive relevance of TLS in breast cancer, the barriers limiting its clinical translation, and key priorities for future research. Overall, TLS status represents a promising but context-dependent biomarker and potential therapeutic intermediate whose interpretation requires standardized classification and a deeper mechanistic understanding.

## 1 Introduction

Breast cancer (BC) is the most frequently diagnosed malignancy and one of the leading causes of cancer-related mortality among women worldwide (1, 2). Despite substantial advances in early detection and systemic therapies, tumor recurrence, metastasis, and therapeutic resistance continue to limit long-term clinical outcomes. Increasing evidence indicates that these processes are not solely determined by tumor-intrinsic factors but are also critically shaped by the tumor microenvironment (TME), which comprises a dynamic network of stromal and immune components (3–5).

Tertiary lymphoid structures (TLS) have emerged as important immunological features of the TME. TLS are ectopic lymphoid aggregates that develop in non-lymphoid tissues under chronic inflammatory conditions, including cancer. Functionally, TLS may support local antigen presentation, T-cell priming or reactivation, and B-cell activation and maturation (6), thereby serving as sites of adaptive immune coordination within tumors. In breast cancer, TLS have been observed across multiple molecular subtypes, but their significance is highly context dependent.

Nevertheless, the role of TLS in breast cancer remains incompletely understood and, in some settings, controversial. While many studies have linked TLS to improved prognosis and treatment response, others have reported more complex or less favorable associations (7–10). These discrepancies likely reflect differences in TLS maturation, spatial localization, and immune composition as well as variations in study design and assessment methods (11).

Building on previous reviews, this article specifically emphasizes TLS developmental staging, breast cancer-specific heterogeneity, mechanistic integration, and translational challenges that currently limit its clinical implementation. We provide a structured overview of the TLS architecture, formation, classification, functional roles, and clinical relevance in breast cancer.

## 2 Structural organization, developmental stages, and maturation relevance of TLS in breast cancer

### 2.1 Core structural components of TLS

Tertiary lymphoid structures (TLS) are ectopic, non-encapsulated lymphoid aggregates that arise in chronically inflamed tissues and partially recapitulate the architecture of secondary lymphoid organs (12). Rather than representing nonspecific lymphocytic infiltrates, TLS are spatially organized immune niches composed of adjacent T-cell and B-cell areas supported by stromal and vascular elements that enable local adaptive immune responses (13).

The T-cell zone typically contains CD4+ and CD8+ T cells, together with mature dendritic cells, particularly DC-LAMP+ antigen-presenting cells, which may support local T-cell priming or reactivation. Regulatory T cells may also accumulate in this compartment (14). The B-cell zone forms follicle-like structures, and in more developed TLS, may contain a germinal center (GC) with proliferating B cells, a CD21+ follicular dendritic cell (FDC) network, and follicular helper T (Tfh) cells that support B-cell activation, class-switch recombination, and differentiation (15, 16). High endothelial venules (HEVs), characterized by peripheral node addressin (PNAd) expression, are a key feature of bona fide TLS and facilitate the recruitment of naïve and central memory lymphocytes through interactions with L-selectin (17). Together, these cellular, stromal, and vascular components distinguish TLS from diffuse immune cell infiltration.

### 2.2 Developmental stages of TLS

TLS are dynamic structures that exist along a maturation continuum (**Table 1**). Current evidence supports three major developmental states: early TLS (eTLS), primary follicle-like TLS (pTLS), and mature TLS (mTLS; secondary follicle-like TLS). These stages are histologically and functionally distinct, although the classification criteria have not yet been fully standardized across studies (13).

**Table 1:** Classification of TLS in breast cancer according to developmental stage and potential significance.

TLS category	Structural features	Representative markers	Potential significance	Key limitation
Early TLS (eTLS)	Dense lymphoid aggregates with limited organization; no FDC network or germinal center	CD20+CD21−CD23−	Reflects early immune-cell recruitment and local immune aggregation	May be difficult to distinguish from nonspecific lymphoid aggregates
Primary follicle-like TLS (pTLS)	Clearer T-cell/B-cell segregation; emerging follicular organization; no germinal center	CD20+CD21+CD23−	Suggests increasing immune coordination and partial lymphoid organization	Classification criteria are not fully standardized
Mature/secondary follicle-like TLS (mTLS/sTLS)	Organized T-cell and B-cell compartments, FDC network, HEVs, and germinal center	CD20+CD21+CD23+; PNAd+	More likely to support coordinated adaptive immunity and associate with better outcome or treatment response	Maturity definitions vary across studies
Intratumoral TLS	Located within tumor bed or tumor-associated stroma	Morphology plus T/B-cell markers	May reflect more direct immune engagement with tumor cells	Anatomical definitions differ across studies
Peritumoral TLS	Located at the invasive margin or adjacent stromal tissue	Morphology plus T/B-cell markers	May indicate organized immune responses at the tumor boundary and show prognostic relevance in some cohorts	Not necessarily biologically equivalent to intra-tumoral TLS
Immunoregulatory TLS-like aggregates	TLS or lymphoid aggregates enriched with suppressive immune populations	Treg-associated or suppressive immune markers when assessed	May reflect restrained or dysfunctional local immune states	Composition is not routinely assessed in most studies

eTLS consists of dense lymphoid aggregates containing B cells and T cells but lacks FDC networks and germinal centers. Wang et al. defined eTLS as CD20+CD21−CD23− aggregates in TNBC (18). pTLS showed clearer segregation of B-cell and T-cell areas and the emergence of an FDC network, but still lacked a GC. In the same study, these were defined as CD20+CD21+CD23−. The most developed stage, mTLS, contains organized T-cell and B-cell compartments, FDCs, HEVs, and CD23+ GC and was classified as CD20+CD21+CD23+. Importantly, TLS maturity varies across breast cancer settings. In breast cancer brain metastases, most TLS exhibit features of eTLS, with only rare pTLS and no observed GC-containing mTLS.

### 2.3 Functional and clinical relevance of TLS maturation

TLS maturation is not merely a morphological feature but also a functionally important variable. GC formation is generally regarded as a hallmark of mTLS because it reflects the acquisition of a more complete local adaptive immune program, including B-cell proliferation, somatic hypermutation, affinity maturation, and plasma cell differentiation, together with more organized T cell–DC interactions. Accordingly, mTLS are more likely than poorly organized lymphoid aggregates to support coordinated humoral and cellular antitumor immunity (19).

In breast cancer, TLS maturity appears to provide more informative biological and clinical signals than does TLS abundance alone. In TNBC, mTLS correlated more strongly with neoadjuvant treatment response and prognosis, whereas in breast cancer brain metastases, a higher TLS density could still be observed in lesions where most TLS remained immature (20, 21). These findings suggest that TLS abundance and maturity are related but distinct variables.

## 3 Mechanisms driving TLS formation and maturation

**Figure 1:**
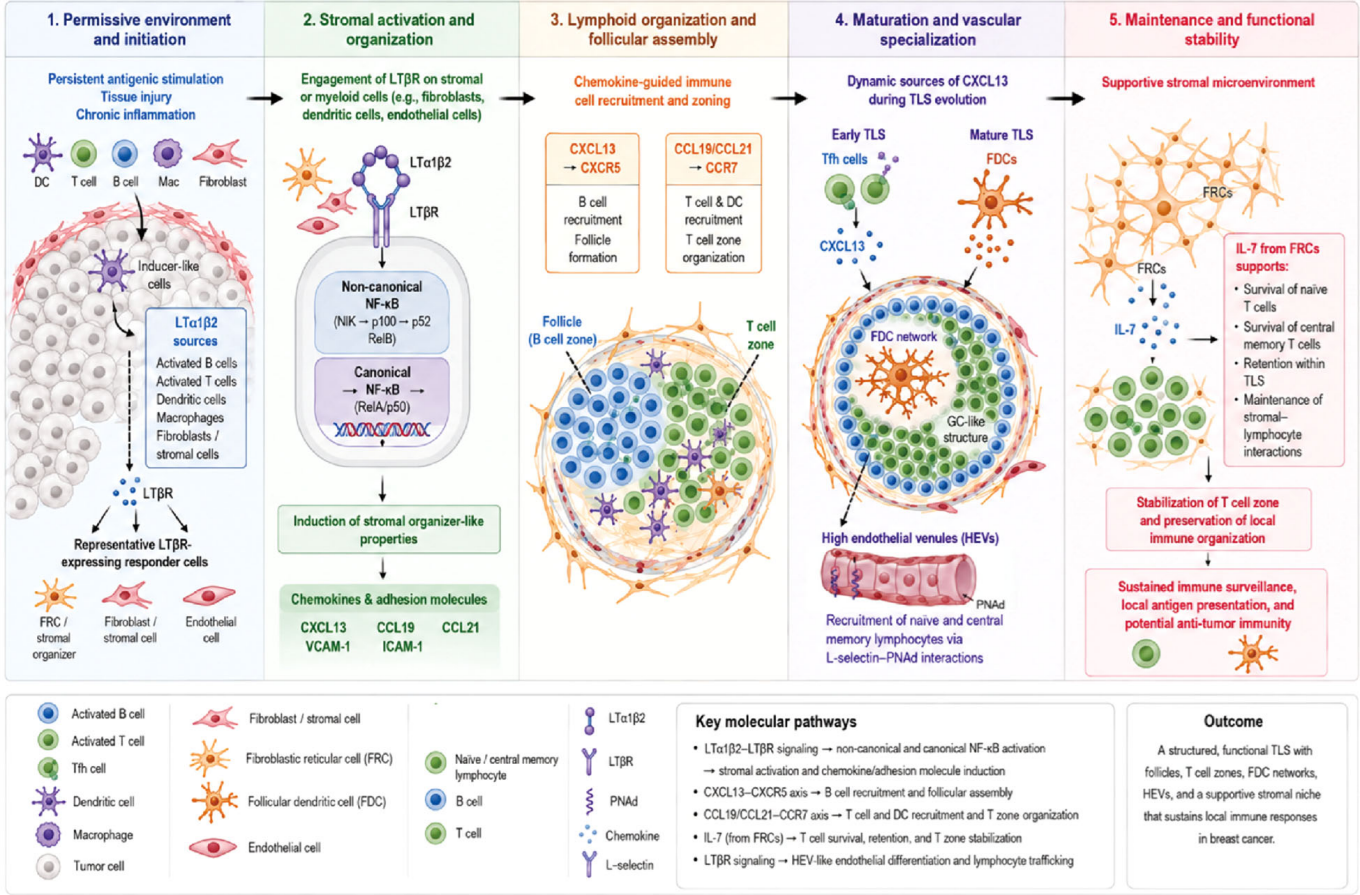
Mechanistic framework of tertiary lymphoid structure formation, maturation, and functional maintenance in breast cancer. Persistent antigenic stimulation, tissue injury, and chronic inflammation create a permissive microenvironment for tertiary lymphoid structure (TLS) initiation. Tumor tissue is depicted as the permissive microenvironmental context, whereas representative LTβR-expressing responder cells are shown separately, including FRC/stromal organizer cells, fibroblast/stromal cells, and endothelial cells. Activated immune and stromal cells provide inducer-like signals, including LTα1β2–LTβR signaling, which promote stromal organizer-like activation and the expression of chemokines and adhesion molecules such as CXCL13, CCL19, CCL21, VCAM-1, and ICAM-1. These cues coordinate immune cell recruitment and spatial organization, with the CXCL13–CXCR5 axis supporting B-cell follicular assembly and the CCL19/CCL21–CCR7 axis promoting T-cell and dendritic cell organization. During maturation, follicular dendritic cell networks, germinal center-like structures, and PNAd+ high endothelial venules contribute to lymphoid organization, lymphocyte trafficking, and functional maintenance. Together, these processes support the development of structured TLS that may sustain local adaptive immune responses and antitumor immunity in breast cancer.

TLS formation in breast cancer should not be regarded as simple recapitulation of embryonic secondary lymphoid organogenesis. Rather, they represent an ectopic lymphoid-organizing process driven by persistent antigenic stimulation, tissue injury, and chronic inflammation, in which local stromal, immune, and endothelial cells cooperatively establish a lymphoid-like niche within tumor-associated tissues. In more immunogenic contexts, such as subsets of triple-negative and HER2-positive breast cancer, ongoing inflammatory cues and pre-existing immune infiltration may create a permissive environment for TLS initiation (22). However, these permissive conditions alone are unlikely to be sufficient to drive TLS maturation (**Figure 1**).

Among the best-characterized organizational pathways, the LTα1β2-LTβR axis is considered to be a central regulator of TLS formation and maintenance. Importantly, the upstream cellular sources of LTβR-activating signals appear to be tissue- and stage-specific (17). Unlike the classical lymphoid tissue inducer (LTi)-dependent program of secondary lymphoid organ development, tumor-associated TLS may be initiated by multiple cell types capable of providing inducer-like signals, including activated B cells, T cells, dendritic cells, macrophages, and inflammation-reprogrammed fibroblasts or stromal cells. LTi-like cells may still contribute to some tumor settings, but current evidence does not support the description of tumor TLS as being uniformly or linearly dependent on canonical LTi cells. In breast cancer, activated B cells and tumor-associated fibroblasts have been proposed as sources of LTβR-activating ligands that functionally substitute classical LTi-like activity (23).

At the molecular level, engagement of LTβR on stromal or myeloid cells by membrane-bound LTα1β2 predominantly activates the non-canonical NF-κB pathway, with an additional contribution from canonical NF-κB signaling (24). This program promotes the acquisition of lymphoid tissue organizer-like stromal properties and induces chemokines and adhesion molecules required for immune cell recruitment, retention, and spatial organization, including CXCL13, CCL19, CCL21, VCAM-1, and ICAM-1 (25). Functionally, these signals are not interchangeable. The CXCL13-CXCR5 axis is more closely linked to B-cell recruitment, follicle formation, and subsequent germinal center-like reactions, whereas the CCL19/CCL21-CCR7 axis is more directly involved in T-cell and dendritic cell recruitment and organization in the T-cell zone (25). Thus, CXCL13 is better understood as a key chemokine for follicular assembly and TLS maturation than as a universal initiating factor.

Beyond the induction and organizational phases, the persistence and functional stability of TLS depends on the establishment of a supportive stromal microenvironment. Fibroblastic reticular cells (FRCs), as key components of stromal remodeling, not only provide structural scaffolding, but also contribute to the maintenance of a lymphoid-like niche (26). In this context, stromal-derived homeostatic cytokines, particularly IL-7 (24), play a critical role in sustaining lymphocyte survival, especially that of naive and central memory T cells, and in promoting their retention within developing TLS. By supporting T-cell viability and maintaining stromal–lymphocyte interactions, IL-7 contributes to the stabilization of the T-cell zone, thereby supporting the structural integrity and persistence of the local immune organization (27).

In addition to the stromal and endothelial compartments, immune cells may actively participate in TLS induction and maturation. In breast cancer, CXCL13 production appears to be dynamically regulated during TLS evolution. Tfh cells may serve as an early source of CXCL13 during initial follicular organization, whereas more mature TLS may increasingly rely on FDCs as a dominant local source (28). This shift supports a stepwise model of TLS development, progressing from inflammatory recruitment to local organization and ultimately follicular maturation. Accordingly, the emergence of FDC networks and GC-like structures reflects a more advanced level of immune organization and distinguishes mTLS from random lymphoid aggregates (29).

HEVs require precise mechanistic interpretation. HEVs are not simply passive consequences of established TLS, nor should they be equated with TLS themselves. Rather, they represent a key component of TLS-associated vascular remodeling that facilitates the entry of circulating naïve and central memory lymphocytes into inflamed tissues, largely through PNAd-dependent interactions with L-selectin (30). LTβR signaling can promote the acquisition of HEV-like endothelial features, and HEV formation is increasingly regarded as both a hallmark of TLS maturation and a vascular hub linking the local immune organization to sustained lymphocyte supply and functional maintenance (31).

Taken together, TLS formation in breast cancer is best understood as a dynamic and context-dependent process involving stromal reprogramming, chemokine-guided immune recruitment, vascular specialization, and progressive follicular organization (32). Nevertheless, much of the current mechanistic framework remains extrapolated from broader studies of TLS biology across tumor types, and breast cancer-specific mechanistic validation, particularly across molecular subtypes and disease stages, remains limited.

## 4 Methods for identifying and classifying TLS

Accurate identification of TLS remains challenging in breast cancer because diffuse lymphocytic infiltration, focal immune aggregates, and highly organized lymphoid structures may coexist within the same tumor, but are not biologically equivalent. Therefore, TLS assessment should not rely solely on lymphocyte density but also on spatial organization, compartmentalization, and maturation. In practice, reliable TLS evaluation requires integration of morphology with immunophenotypic and, increasingly, molecular information.

### 4.1 Histopathology and conventional immunohistochemistry

Hematoxylin and eosin (H&E) staining remains the most accessible method for initial TLS assessment and is useful for identifying dense lymphoid aggregates, their distribution, and follicle-like architecture within the tumor stroma or at the invasive margin (12). Whole-section evaluation is particularly informative in primary surgical specimens because it permits assessment of TLS location relative to the tumor bed and invasive front. By contrast, limited core biopsies may under-sample focal TLS. Post-treatment, recurrent, or metastatic samples should also be interpreted with caution, because prior therapy and tissue context may alter both TLS abundance and maturation.

Conventional immunohistochemistry (IHC) improves classification by characterizing the key cellular and stromal components. Commonly used markers include CD3 for T cells, CD20 for B cells, CD21 or CD23 for follicular structures and GC-related maturation, and PNAd for HEVs (33). In breast cancer, marker combinations, such as CD20/CD21/CD23, have been used to distinguish eTLS, pTLS, and GC-containing mTLS. However, no universally accepted marker panel or minimum definition exists. At a minimum, TLS identification should go beyond lymphocyte density and include evidence of structural organization, ideally with discernible T-cell/B-cell segregation and, when feasible, features of maturation, such as FDC networks or GCs. Importantly, not all TLS display every mature feature at the time of sampling; early TLS may lack HEVs or GC formation despite representing biologically relevant lymphoid organization.

### 4.2 Multiplex imaging and molecular approaches

Multiplex immunofluorescence (mIF) and related imaging approaches provide greater resolution than single-marker IHC by allowing the simultaneous visualization of multiple immune and stromal components within the same section (34). These methods are particularly useful for identifying compartmentalized TLS architectures, including T-cell zones, B-cell follicles, Tfh cells, plasma cells, FDC networks, and HEV-associated vasculature (35) (36). In breast cancer, where TLS heterogeneity is substantial, multiplex imaging is especially informative for assessing TLS composition and maturation state. Its limitations include cost, technical complexity, and limited standardization, which restrict routine clinical use.

Transcriptomic methods provide a scalable way to infer TLS enrichment in large datasets and have shown value in predicting treatment responses in breast cancer (37). However, gene expression signatures may identify immune-rich tumors without confirming the presence of structurally organized TLS. Therefore, molecular enrichment should not be considered equivalent to histologically mature TLS. Spatial transcriptomics may help bridge this gap by combining molecular profiling with tissue architecture; however, it remains costly and is not yet routine in clinical pathology. Thus, transcriptomic approaches are best interpreted alongside morphological or imaging-based evidence, whenever possible.

### 4.3 Current challenges in TLS classification

A major unresolved issue is the lack of standardized criteria for TLS definition and classification. Across studies, TLS has been scored using different anatomical thresholds, marker combinations, and maturity criteria, which limits direct comparison and contributes to inconsistent conclusions regarding its prognostic or predictive value. For breast cancer, future progress will require reproducible and clinically feasible classification frameworks that distinguish diffuse immune infiltration from organized lymphoid structures and explicitly state the disease setting and specimen type being assessed. Interobserver variability, sampling bias, and integration into routine pathology workflows remain major barriers to clinical implementation.

## 5 TLS heterogeneity in breast cancer

TLS in breast cancer should not be regarded as a uniform immune entity. Rather, they display substantial heterogeneity in their maturation state, spatial localization, and cellular composition, and these differences influence both their biological functions and clinical significance.

### 5.1 Heterogeneity by maturity

TLS maturity is one of the most important sources of heterogeneity. Breast cancer-associated TLS range from poorly organized eTLS to pTLS and GC-containing mTLS. Increasing evidence suggests that TLS maturity may be more informative than TLS abundance alone when evaluating the prognosis or treatment response. For example, Song et al. reported that the density of TLS-associated high endothelial venules (HEVs) correlated with pathological complete response (pCR) and survival, although HEV density should be interpreted as a surrogate marker of TLS maturation rather than as a direct equivalent of TLS maturity itself. However, because the criteria for defining mature versus immature TLS are not yet fully standardized across studies, caution is required when comparing the results across cohorts.

### 5.2 Heterogeneity by location

TLS also differ according to their anatomical location. Intratumoral TLS are lymphoid aggregates located within the tumor parenchyma, embedded among tumor cell nests, or within the intratumoral stroma, and are therefore in more direct contact with malignant cells. In contrast, peritumoral TLS are distributed outside the main tumor mass, most commonly at the invasive margin or within adjacent stromal or non-malignant tissues, without direct integration into tumor cell nests.

However, these spatial categories are unlikely to be biologically equivalent. Intratumoral TLS may more directly reflect immune activity within the tumor bed, whereas peritumoral TLS may represent immune organization at the invasive margin or in the adjacent stromal tissue. Nevertheless, their definitions and pathological assessments vary across studies. Boissière-Michot et al. reported that peritumoral TLS was associated with improved relapse-free survival in TNBC, whereas Sofopoulos et al. found that peritumoral TLS was associated with poorer survival in invasive ductal breast carcinoma (38). These apparently discordant observations suggest that the biological impact of the TLS location is likely context-dependent rather than uniform. TLS distribution may also be influenced by organ-specific immune environments. Differences between primary breast tumors and metastatic lesions indicate that local tissue context may influence not only TLS abundance, but also their maturation and functional state. Lee et al. further showed that TLS distribution varies across metastatic sites and is associated with TIL levels and survival (39).

### 5.3 Heterogeneity across breast cancer subtypes and immune composition

Breast cancer subtype is another major determinant of TLS heterogeneity. Triple-negative and HER2-positive tumors generally exhibit greater immunogenicity, denser immune cell infiltration, and a higher prevalence of TLS than luminal tumors (40). In TNBC, TLS presence and maturity have frequently been associated with stronger immune activation, a better response to neoadjuvant therapy, and a more favorable prognosis, particularly when mTLS are present (41). Similar associations between TLS and treatment outcomes have been reported in some HER2-positive cohorts. In contrast, luminal tumors tend to exhibit weaker immune infiltration overall, and the prevalence, maturity, and clinical significance of TLS appear less consistent.

In addition to subtype-level differences, TLS composition may also vary across breast cancer settings. TLS enriched in Tfh cells, B cells, mature dendritic cells, and cytotoxic T cells are more likely to support productive antitumor immunity (42), whereas TLS-like aggregates enriched in Treg cells or other suppressive immune populations may reflect a more restrained local immune state (43, 44). Such compositional variation may contribute to inter-study heterogeneity and influence the context-dependent interpretation of the TLS in breast cancer.

Taken together, TLS heterogeneity in breast cancer is shaped by the maturation state, anatomical location, tumor subtype, and immune cellular composition, all of which should be considered when interpreting their biological and clinical significance.

## 6 Functional roles of TLS in breast cancer: antitumor, pro-tumor, and context-dependent effects

TLS are generally regarded as sites of local immune organization in tumors; however, their functional significance in breast cancer is not uniformly beneficial. Instead, TLS may support antitumor immunity, sustain immunoregulatory programs, or exert context-dependent effects depending on their maturation state, cellular composition, spatial location, and disease setting.

### 6.1 Antitumor immune functions of TLS

In their more organized and mature forms, TLS may promote local adaptive immunity by supporting coordinated interactions between dendritic cells, T cells, B cells, and stromal elements (42). Mature dendritic cells facilitate antigen presentation and T-cell activation, whereas follicular structures and GC-like reactions support B-cell proliferation, affinity maturation, class-switch recombination, and differentiation into plasma cells and memory B cells (19, 45). In this setting, TLS may function as a local immune hub that enhances both the cellular and humoral antitumor responses.

In breast cancer, particularly triple-negative disease, mTLS have been associated with stronger immune activation, increased immunoglobulin-related transcription, enhanced cytotoxic T-cell activity, and improved response to neoadjuvant therapy. These observations support the view that well-organized TLS can contribute to effective tumor-specific immune surveillance.

### 6.2 Immunoregulatory and potentially pro-tumor effects

TLS should not be assumed to be uniformly protective. Their cellular composition may include regulatory or suppressive populations that inhibit effective antitumor immunity. Regulatory T cells can accumulate within TLS and inhibit T-cell activation (46, 47), whereas regulatory B-cell-like populations or suppressive myeloid cells may further shift the local immune balance toward immune restraint rather than immune elimination (48, 49). In some breast cancer studies, TLS-associated immune aggregates enriched in immunoregulatory cell populations have been linked to less favorable outcomes, indicating that the mere presence of TLS does not necessarily imply effective antitumor immunity (50).

Thus, TLS may support qualitatively distinct immune states: in some contexts, they amplify effector immunity, whereas in others, they may reflect or sustain a partially suppressed immune microenvironment.

### 6.3 Context-dependent interpretation of TLS function

The functional significance of TLS in breast cancer should be interpreted in this context. mTLS enriched in Tfh cells, B cells, mature dendritic cells, and cytotoxic T cells are more likely to reflect productive local immune activation, whereas poorly organized or immunoregulatory TLS-like aggregates may have limited functional value or may even be associated with immune restraint (51). Differences in molecular subtypes, anatomical sites, disease stages, and TLS assessment methods may all contribute to the variable associations reported across studies.

## 7 Clinical relevance of TLS in breast cancer: prognostic value, predictive utility, and therapeutic potential

Because TLS reflect localized immune organization within the tumor microenvironment, they have attracted increasing interest as a potential prognostic biomarker, predictor of treatment response, and possible therapeutic intermediate in breast cancer. However, their clinical relevance should be cautiously interpreted.

### 7.1 Prognostic value

A substantial body of evidence suggests that TLS in breast cancer is often associated with more favorable clinical outcomes (52), particularly in immunogenic subtypes such as triple-negative and HER2-positive diseases (53, 54). In these settings, TLS presence or enrichment has been linked to prolonged survival, a lower risk of progression, and a more inflamed tumor microenvironment. Increasing evidence also suggests that TLS maturity may be more informative than TLS abundance alone, as GC-containing mTLS are more likely to reflect coordinated and functionally active local immune responses. In addition, Bertucci et al. reported that TLS-related gene signatures were associated with pathological complete response (pCR) and with greater benefit from pembrolizumab-containing therapy, further supporting the clinical relevance of TLS-associated immune organization (55).

However, the prognostic value was not uniform. TLS impact may vary according to molecular subtype, anatomical location, and disease stage, and some studies have reported more complex or less favorable associations in specific settings (56, 57). Thus, TLS status is a promising prognostic biomarker, but its clinical interpretation remains context dependent. Representative studies evaluating the prognostic and predictive significance of TLS in breast cancer are summarized in **Table 2**.

**Table 2:** Representative studies evaluating the prognostic and predictive significance of tertiary lymphoid structures in breast cancer.

Author	Year	Breast Cancer Subtype	No. of patients	Identification of TLS	Grouping methods	Survival outcome	Finding	Limitation
François Bertucci (55)	2023	HER2-negative	248	Coppola’s 12‐chemokine signature	Median: TLS-high VS TLS-low	pCR	Better pCR	Largely retrospective
Liye Wang (37)	2023	All subtypes	866	TCGA 9-chemokine signature	TLS-high VS TLS-low	OS	Longer OS	No histological validation
Xia Liu (58)	2017	HER2-positive and HER2-negative	248	H&E and IHC(CD3, CD20)	Absent VS Present	DFS	Longer DFS	Outdated treatment era
Lixia Zeng (50)	2023	All subtypes	198	H&E,FOXP3, CD163,CD4 and CD4/CD8	TLS-positive VS TLS-negative	DFS	Shorter DFS	Semi-quantitative TLS assessment; limited reproducibility
Qing Wang (33)	2023	NA	14	single-cell transcriptome sequencing	Median: TLS-high VS TLS-low	NA	Better prognosis and neoadjuvant therapy response	No external validation
Michael Sofopoulos (38)	2019	Invasive ductal BC	167	H&E, IHC CD3, CD4, CD20, CD23, CD31, CD163	No, Low, Moderate, Abundant	DFS; OS	Shorter DFS and OS	Non-standardized TLS scoring
Dominique Yuan Bin Seow (59)	2020	TNBC	269	H&E, IHC CD38, CD20	TLS-positive VS TLS-negative	NA	Favorable prognostic features	Limited functional validation
In Hye Song (41)	2017	TNBC	108	H&E, IHC CD3, CD8, CD20	No, Low, Moderate, Abundant	DFS	Better pCR and longer DFS	Small single-center cohort
Miseon Lee (39)	2019	Metastasis BC	335	H&E	Absent VS Present	OS	Better OS	Potential confounding bias
Jinyuan Gu (54)	2023	Locoregional recurrent BC	112	H&E, IHC CD3, CD4, CD8, CD19, CD38 and CD68	Median: TLS-high VS TLS-low	PFS	Longer PFS	Non-standardized assessment
Xiaonan Zhang (60)	2025	NA	9551	AI-assisted TLS signature derived from 4 key TLS-associated genes	High-risk VS Low-risk	OS; PFS	Higher TMB and poorer prognosis; Greater effectiveness of chemotherapy and poorer efficacy of immune checkpoint inhibitors	Computational TLS; Overfitting risk
Xiaoxiao Wang (61)	2024	TNBC	92	H&E, IHC CD3, CD20 Spatial Transcriptomics 30-gene TLS	High expression VS Low expression	DRFS; iBCFS; PFS	Better DRFS and iBCFS; Longer PFS and better radiological response	No standardized TLS definition
Florence Boissière-Michot (62)	2024	TNBC	397	H&E, IHC CD3, CD20	(PT-TLS): None, Low, Moderate, Abundant.IT-TLS, m-TLS: Present VS Absent	RFS	PT-TLS: Longer RFS; PT-TLS and higher TILs, IT-TLS, m-TLS:NA	Single-center; Semi-quantitative scoring

pCR, pathological complete response; PFS, progression-free survival; TNBC, triple-negative breast cancer; DRFS, Distant Relapse-Free Survival; iBCFS, Invasive Breast Cancer-Free Survival; PT-TLS, peritumoral TLS; IT-TLS, Intratumoral TLS; RFS, Relapse-Free Survival; NA, Not available.

### 7.2 Predictive utility for therapy response

TLS status has also emerged as a potential predictive biomarker for treatment response (13). Several studies on breast cancer have shown that TLS enrichment is associated with an improved pathological response to neoadjuvant therapy, particularly in triple-negative disease (59). More recently, TLS-related transcriptional signatures have shown potential for identifying patients with HER2-negative breast cancer who may derive greater benefits from neoadjuvant treatment and immune checkpoint blockade. These observations support the concept that TLS may mark a pre-existing or therapy-responsive adaptive immune microenvironment (63).

However, its predictive application is still at an early stage. Most available evidence is retrospective, prospective validation remains limited, and it is still unclear whether histologically defined TLS, TLS maturity, or transcriptomic TLS signatures will be most suitable for clinical use. Timing is also important because pretreatment of primary tumor samples may not fully reflect immune remodeling in residual, recurrent, or metastatic disease. Therefore, TLS are not yet ready for routine treatment stratification of breast cancer.

### 7.3 Therapeutic potential and barriers to translation

Beyond biomarker use, TLS are increasingly being considered potential therapeutic targets or intermediates. In principle, strategies that promote TLS formation, enhance their maturation, or improve their functional quality could strengthen local antitumor immunity and increase treatment responsiveness, particularly in poorly inflamed tumors (64, 65). Potential approaches include promoting HEV formation, strengthening chemokine-guided lymphoid organization, or combining TLS-favoring microenvironmental modulation with immune checkpoint blockade. However, this concept remains largely unexplored in breast cancer. It remains unclear how to induce durable and functionally beneficial TLS while avoiding the generation of ineffective or immunoregulatory immune structures.

Multiple barriers currently limit its clinical translation. There is no universally accepted standard for TLS definition, scoring, or maturity classification, and different assessment methods capture the overlapping but non-identical dimensions of TLS biology. Accordingly, before TLS can be incorporated into routine biomarker-guided management, standardized classification systems, reproducible pathology workflows, and subtype-specific prospective validation are required.

## 8 Current controversies, evidence limitations, and future directions

Despite growing interest in TLS as a biomarker and potential modulator of antitumor immunity, several important controversies remain regarding its role in breast cancer. A major unresolved issue is that TLS are often discussed as biologically uniform, whereas current studies assess structures that may differ substantially in maturation state, spatial location, and immune composition. Consequently, conflicting conclusions across studies may reflect differences in TLS definitions and classifications, rather than true biological contradictions. In particular, the distinction between diffuse lymphoid aggregates, eTLS, and GC-containing mTLS has not been applied consistently, which limits both biological interpretation and inter-study comparability.

The quality of evidence remains uneven. Much of the current literature is retrospective, based on single-center cohorts, and enriched for selected subtypes or disease settings (66). Sample size, treatment background, endpoint definition, and tissue source vary substantially across studies, making it difficult to determine whether the reported associations are robust, generalizable, and independent of confounding clinicopathological factors. In addition, primary tumors, post-neoadjuvant residual disease, recurrent lesions, and metastatic sites are often analyzed together or compared indirectly, although TLS in these settings may not be biologically equivalent. These limitations reduce reproducibility and weaken the current evidence for the TLS-guided interpretation of breast cancer.

Methodological inconsistencies also remain a major barrier. Studies differ in their use of H&E staining, immunohistochemistry, multiplex imaging, and transcriptomic signatures, each of which captures overlapping but non-identical aspects of TLS biology. Without harmonized definitions, standardized scoring systems, and clearer maturity criteria, translation into routine pathology workflows and treatment decision-making remain difficult.

Therefore, future studies should focus on several priorities. First, standardized classification frameworks are needed to distinguish diffuse immune infiltration from organized TLS and reproducibly assess maturation, compartmentalization, and GC status. Second, prospective and subtype-specific studies are required to determine when TLS provides independent prognostic or predictive information beyond established immune biomarkers, such as TILs and PD-L1 (67). Third, breast cancer-specific mechanistic studies are needed to clarify how stromal programs, vascular remodeling, and local immune composition regulate TLS formation, maintenance, and function across primary tumors, residual diseases, and metastatic sites. Addressing these issues will be essential not only for improving TLS-based biomarker development but also for determining whether TLS can be manipulated therapeutically in a safe and clinically meaningful way.

## 9 Conclusion

Tertiary lymphoid structures are increasingly recognized as key components of the breast cancer immune microenvironment, yet their biological and clinical significance cannot be reduced to a simple TLS-positive or TLS-negative classification. Current evidence indicates that the relevance of TLS depends on their maturation state, spatial distribution, cellular composition, and disease context. In particular, GC-containing mTLS are more likely to reflect productive local adaptive immunity, whereas immature or immunoregulatory TLS-like aggregates may have more limited or context-dependent significance. Standardized classification frameworks, reproducible pathology workflows, and stronger breast cancer-specific validation are therefore essential for clarifying their biomarker value and therapeutic relevance. Deeper mechanistic studies may further inform future strategies for TLS-directed immune modulation.
